# Tracheal Trifurcation Associated With Esophageal Atresia

**Published:** 2010-12-01

**Authors:** Yogesh Kumar Sarin

**Affiliations:** Department of Pediatric Surgery, Maulana Azad Medical College New Delhi, India

**Keywords:** Esophageal atresia, Tracheo-esophageal fistula, Tracheal bronchus, Tracheal trifurcation

## Abstract

We report a newborn with esophageal atresia (EA) in whom right tracheal bronchus (TB) and a tracheal diverticulum were identified intra-operatively. The right TB was further confirmed on MRI scan performed post-operatively. Such a tracheal trifurcation associated with EA has not been reported hitherto from Indian subcontinent.

## INTRODUCTION

VACTERL, CHARGE, and SHISIS are well known non-syndromic associations of EA that are mentioned in neonatal surgery text books. However, other than tracheomalacia, association of tracheo-bronchial anomalies with EA is not well highlighted. Tracheal trifurcation or aberrant origin of the upper lobe bronchus, especially of the right side (also known as right TB) is occasionally seen in association with EA. The incidence of right TB in patients of EA is quoted around 4%, although a series had reported an incidence of 37.5% [1-3].

About 1/5th of fetal rats with EA induced by adriamycin had a right TB [4]. Conversely, 11% of patients with TB in series of children aged 1 day to 54 months (mean 17 months) had EA [5]. TB has been reported with VACTERL association on at least three previous occasions [5-7].

We are reporting a newborn having EA in association with right TB along with a brief review of the literature. 

## CASE REPORT

A 1-day-old male neonate weighing 2.1kg was born to a 21-year-old primigravida mother at full term. He was a product of non-consanguineous marriage and delivered by normally. He was brought with classical features of EA. The pregnancy was unremarkable other than polyhydramnios that was diagnosed 3 days prior to the delivery. His abdomen was scaphoid. There were no obvious associated anomalies. Nasogastric tube could not be passed into the stomach. Plain x-ray abdomen was gasless suggestive of isolated EA with no distal tracheo-esophageal fistula (TEF). There were 13 pairs of ribs and no vertebral anomalies were noted. Echocardiography was reported as normal. Ultrasonography of the abdomen revealed moderate left hydronephrosis.

A staged repair of EA was planned. When laparotomy was performed for abdominal esophagostomy, the stomach was found to be distended. It was then decided to proceed with a right thoracotomy to rule out a blocked distal TEF. The thoracotomy revealed a long-gap EA with the proximal esophageal pouch hardly traversing the thoracic inlet, and right-sided aortic arch. An 8 mm long blindly-ending, distensible tracheal diverticulum was seen to arise from the right lateral wall of the trachea that was excised (Fig. 1). The right upper lobe bronchus was seen arising from the carina (Fig. 2). Thorax was then closed. Cervical esophagostomy and abdominal esophagostomy were fashioned.

**Figure:**
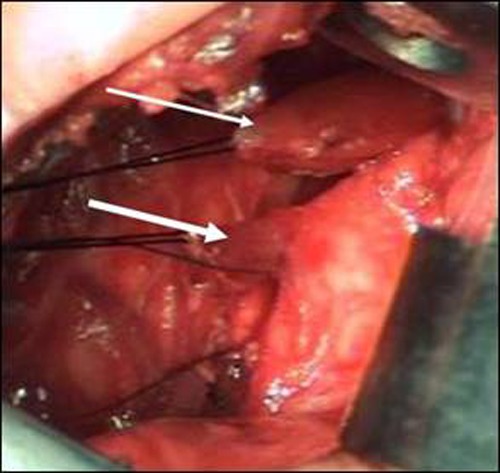
Figure 1: Intra-operative picture showing the proximal esophageal pouch (thin white arrow) and blind tracheal diverticulum (thick white arrow).

**Figure:**
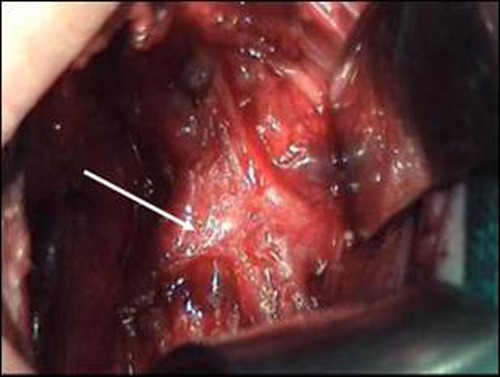
Figure 2: Intra-operative picture showing right TB (white arrow).

Post-operatively, a MRI chest was done that confirmed the diagnosis of right TB (Fig. 3). The histopathology of the excised tracheal diverticulum revealed fibro-collagenous tissue and smooth muscle wall lined with squamous epithelium, ciliated pseudostratified columnar epithelium and gastrointestinal type of endothelium. Few seromucinous glands were seen in the sub-epithelium. No cartilage was found. The post-operative course was uneventful and patient discharged on feeding through abdominal esophagostomy. Sham feeds from mouth were also instituted. 

**Figure:**
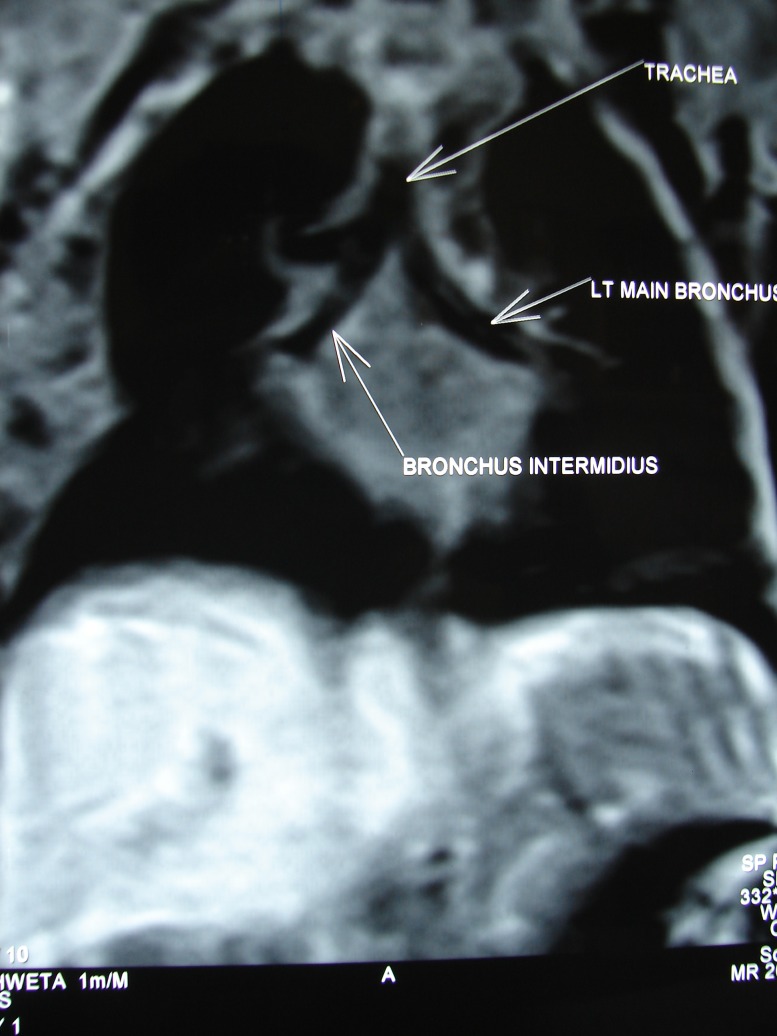
Figure 3: Post-operative MRI showing right upper lobe bronchus originating at the level of the carina.

At 7 month of age, a gastric pull-up was performed. Patient had severe tachycardia both intra-operatively and post-operatively. He had minor anastomotic leak in the neck that healed spontaneously. He was discharged on oral omeprazole and erythromycin that was continued for 6 months. The child has been on regular follow up for last 5 years, and doing well. His somatic growth is in 30th percentile.

## DISCUSSION

TB was described by Sandifort in 1785 as a right upper bronchus originating in the trachea. In the recent literature, the term TB encompasses a variety of bronchial anomalies originating from the trachea or main bronchus and directed to the upper lobe territory. These may be displaced or supernumerary (accessory) [8]. A classification of aberrant bronchi directed to the upper lung lobes is displayed as line-diagram (Fig. 4). By this classification, our patient had displaced right TB. Other authors have described similar anomaly as tracheal trifurcation. Displaced right TB is incidentally the commonest type of bronchial anomaly reported in literature [8,9].

**Figure:**
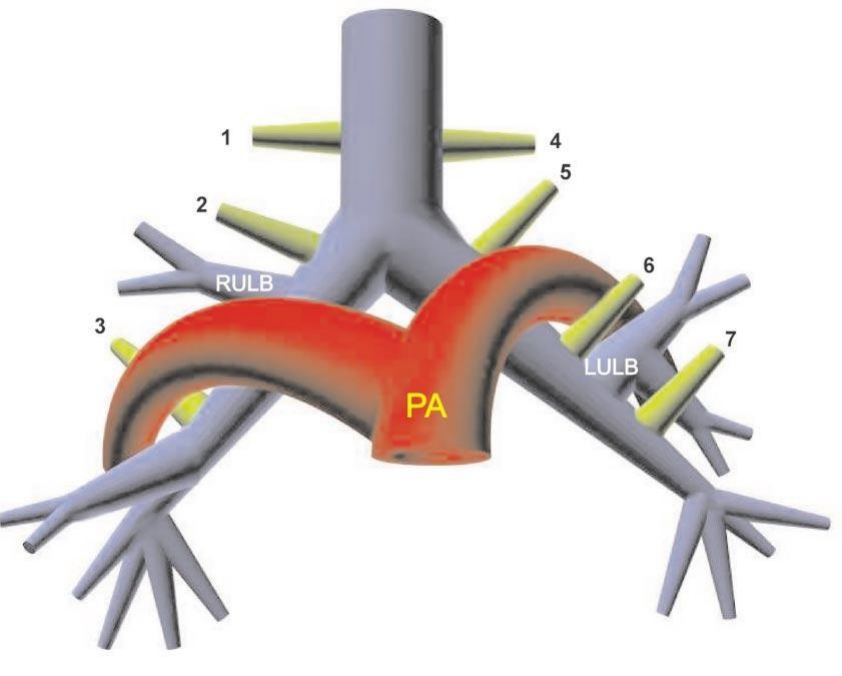
Figure 4: Schematic representation of aberrant bronchi to the upper lobes: prearterial (true right tracheal) (1), preeparterial (right “tracheal”) (2), posteparterial (3), eparterial (true left tracheal) (4), eparterial (left “tracheal”) (5), prehyparterial (6), and posthyparterial (7) bronchi. LULB = left upper lobe bronchus, PA = pulmonary artery, RULB = right upper lobe bronchus. (reproduced with permission of publishers of Radiographics).

The prevalence of TB in normal population is 2% or less. It is 7 times more common on the right side. Almost all the TB reported in association with EA previously have been reported on right side [6,8,10].

Our patient also had a tracheal diverticulum that could either be a blindly-ending supernumerary bronchus or proximal remnant of an aborted distal TEF [11].

Patients with TB are usually asymptomatic. However, TB have been known to cause persistent or recurrent upper-lobe pneumonia, atelectasis or air trapping, and chronic bronchitis, bronchiectasis, focal emphysema, and cystic lung malformations. When associated with VACTERL anomalies, TB has been known to coexist with other tracheo-bronchial anomalies such as tracheo-bronchial stenosis or tracheo-bronchomalacia, further adding to the respiratory complications. Associated rib anomalies have also been reported [5-7,8,10].

Traditional diagnostic radiological modality of TB, the bronchography, has been replaced by virtual bronchoscopy and MRI. TB could be directly visualized on bronchoscopy. A few centers routinely do pre-operative bronchoscopy for all EA patients, when a TB may be incidentally diagnosed by experienced anesthesiologist or neonatal surgeon [1,2]. Rarely, the diagnosis is made in an incidental surgery as in our case. Had we not performed a thoracotomy, and straightaway headed to perform the cervical and abdominal esophagostomies, the diagnosis could have been missed, may be forever.

Different centers have different policies about anesthesia for EA with TEF patients. While most of the anesthetists plan placing an endotracheal tube just above or below the TEF, few prefer doing left bronchus intubation, or using double-lumen tube. Endotracheal intubation in a patient with a TB can cause obstruction of the TB leading to shunting and hypoxemia. Intubation of the TB may result in hypoxia, atelectasis, or both during anesthesia. Similar complications may be encountered postoperatively when EA patient having undergone primary anastomosis is electively ventilated. Recognition of a TB before induction of intubation can be helpful for determining optimal positioning of the endotracheal tube [12].

Most patients with TB can be treated conservatively; however, in symptomatic patients surgical excision of the involved segment is necessary. Though rare, lung cancer arising from TB has been reported [11,13].

It is proposed that precise tracheo-bronchial anatomy should be known in all the patients of EA. It is suggested that in those centers where a preoperative bronchoscopy is not performed for patients of EA, a flexible bronchoscopy may be performed in infancy to rule out associated tracheomalacia and TB.

## Footnotes

**Source of Support:** Nil

**Conflict of Interest:** None declared
